# Scientific Advances in Controlling *Nosema ceranae* (*Microsporidia*) Infections in Honey Bees (*Apis mellifera*)

**DOI:** 10.3389/fvets.2019.00079

**Published:** 2019-03-15

**Authors:** Andre J. Burnham

**Affiliations:** Emory University, Atlanta, GA, United States

**Keywords:** honey bee, *Nosema ceranae*, nosemosis, fumagillin, RNA interference, phytotherapy, colony collapse disorder

## Abstract

Honey bees (*Apis mellifera*) are agriculturally important pollinators that have been recently at risk to severe colony losses. A variety of parasites and pathogens have been linked to colony decline, including the microsporidian parasite *Nosema ceranae*. While fumagillin has been used to control nosemosis in managed honey bee colonies for decades, research shows that this antibiotic poses a toxic threat and that its efficacy against *N. ceranae* is uncertain. There is certainly a demand for a new veterinary medication to treat honey bee colonies infected with *N. ceranae*. In this review, recent scientific advances in controlling *N. ceranae* infections in honey bees are summarized.

## Introduction

Honey bees (*Apis mellifera*) are pollinators with a significant worldwide economic value and are responsible for the pollination of many ecologically and agriculturally important crops (1, 2). Managed honey bee colonies have been in decline for the past several decades, notably in North America (3, 4). This decline is of growing concern on account of the crucial role honey bees play in sustaining human and livestock food sources (2, 5). Colony losses have been linked to pesticide exposure, environmental and migratory stress, and poor nutrition; however, parasite and pathogen infections are likely the leading factors contributing to colony mortality (4, 6–12).

*Nosema ceranae* is an obligate microsporidian intracellular parasite infectious to honey bees (13–15). While *Nosema apis* and *N. ceranae* both parasitize honey bees, *N. ceranae* has geographically outcompeted *N. apis* (16–20). Severe *N. ceranae* infections (nosemosis) can cause bee mortality and have been correlated with colony losses (7, 13, 15, 21–24). *N. ceranae* is also associated with morbid physiological impairments including suppressed immune function, foraging behavior, pheromone and hormone production, and lipid synthesis (25–30).

A spore-forming fungal parasite, *N. ceranae* is transmitted orally via honey, nectar, pollen and bee fecal matter (31). The reproductive cycle of *Nosema* begins shortly after entering the host digestive tract (24, 31, 32). Following germination in the midgut lumen, environmental osmotic pressure causes a specialized organelle called a polar tube to protrude from the spore and inject sporoplasm (infectious material) into the host cytoplasm (32, 33). Meronts then proliferate and mature into primary spores which germinate within the host cell and may auto-infect adjacent cells (24, 33, 34). Primary spores may also develop into fully-formed environmental spores that are released via cell lysis into the midgut lumen (24, 33). Here the reproductive cycle repeats, or free spores are expelled by defecation (31).

As livestock, honey bees require veterinary treatments from beekeepers or agriculturists when infected with parasites or pathogens (35, 36). First isolated in 1949 from the fungus *Aspergillus fumigatus*, fumagillin has been used to treat nosemosis induced by *N. apis* in honey bees for several decades (37). However, recent studies show that this antibiotic may be ineffective against *N. ceranae* infections (38–43). There is also evidence that fumagillin is fairly toxic and causes chromosomal aberrations, carcinogenicity in humans, and alterations to the ultrastructure of hypopharyngeal glands in bees (37). Consequentially, many countries outside of the Americas (including the European Union) have banned fumagillin for agricultural use (MRL; Commission Regulation, EU, 2010, no. 37/2010). There is a significant demand, therefore, for a new medication that safely and effectively treats honey bee colonies infected with *N. ceranae*. Recent molecular, phytotherapeutic, and supplement-based scientific advances that aim to control nosemosis in honey bees (Table 1) are summarized and discussed herein.

**Table 1:** A summary and comparison of anti-*N. ceranae* treatments that have displayed efficacy in previous works.

**Treatment type**	**Bee spore load^**1**^**	**Bee survival^**1**^**	**Hive spore load^**2**^**	**Other effects**	**References**
**SMALL MOLECULES**					
Metronidazole (*in vitro* only)^**^	N/A	N/A	N/A	↓spore viability	(44)
Tinidazole (*in vitro* only)^**^	N/A	N/A	N/A	↓spore viability	(44)
Porphyrin: PP(Asp)_2_	↓	↑	N/A	↓spore viability	(45)
Porphyrin: TMePyP	↓	N/A	N/A	↓spore viability	(45)
Fumagillin analogs^a^^*^	↓	^†^	N/A	N/A	(46)
Fumagillol^*^	↓	^†^	N/A	N/A	(46)
Semisynthetic aspirin^*^	↓	^†^	N/A	N/A	(46)
Enilconazole^*^	↓	^†^	N/A	N/A	(46)
Piperonyl analog^*^	↓	^†^	N/A	N/A	(46)
Thymol^*^	↓	^†^	N/A	N/A	(46)
Formic acid (fumigation)	N/A	N/A	↓	N/A	(47)
Oxalic acid	↓	N/A	N/A	N/A	(48)
Oxalic acid (topical field trial)	N/A	N/A	↓	↑colony survival	(48)
Thymol	↓	↑	N/A	N/A	(49)
Resveratrol	No Effect	↑	N/A	N/A	(49)
Thymol	↓	No Effect	N/A	N/A	(50)
Resveratrol	↓	↑	N/A	N/A	(50)
**RNA INTERFERENCE**					
ADP/ATP transporter RNAi	↓	N/A	N/A	↑response to sucrose	(51)
*ptp3* RNAi	↓	↑	N/A	↑immune expression	(52)
*nkd* RNAi	↓	↑	N/A	↑immune expression	(53)
**EXTRACTS AND SUPPLEMENTS**					
Polysaccharide extracts^*^	↓	↑	N/A	N/A	(54)
Pentadecapeptide BPC 157	N/A	N/A	↓	↓bee midgut lesions; ↑colony strength	(55)
EtOH *L. nobilis* Extract	↓	No Effect	N/A	N/A	(56, 57)
*C. alba* EO extract^**^	↓	↑	N/A	N/A	(58)
Compounds detected in *C. alba* EO extract+^*^	↓	↑	N/A	N/A	(58)
MeOH *A. chilensis* extract	↓	No Effect	N/A	N/A	(59)
MeOH *U. molinae* extract	↓	↑	N/A	N/A	(59)
MeOH *G. avellana* extract	↓	No Effect	N/A	N/A	(59)
MeOH propolis extract	↓	↑	N/A	N/A	(59)
EtOH propolis extract^b^	↓	↑	N/A	N/A	(60)
EtOH propolis extract^c^	↓	↑	N/A	N/A	(61)
BEEWELL AminoPlus	↓	No Effect	N/A	↑immune expression	(62)
Nozevit^d^	N/A	N/A	↓	↑colony strength	(63)
HiveAlive	N/A	N/A	↓	↑colony strength	(64)
**MICROBIAL SUPPLEMENTS**					
Bacterial surfactin	↓	↑	N/A	↓spore viability	(65)
*L. johnsonii* metabolites	N/A	N/A	↓	↑fat bodies per bee; ↑colony strength	(66)
*Bifidobacteria*	↓	N/A	N/A	N/A	(67)
*Lactobacilli*	↓	N/A	N/A	N/A	(67)
*P. apium*	No Effect	↑	N/A	N/A	(68)
*Bacillus* sp.	No Effect	↑	N/A	N/A	(68)
Bactocell	No Effect	↑	N/A	N/A	(68)
Levucell SB	No Effect	↑	N/A	N/A	(68)

*Treatments not delivered orally are labeled as such. An increase is marked by “↑” and a decrease by “↓”. Metrics that were not measured are labeled non-applicable (N/A)*.

^1^Measured in cage/inoculation experiments; ^2^Measured in full colonies;

** As effective as fumagillin according to authors;

* Less effective than fumagillin according to authors;

a Four in-house synthetic fumagillin analogs were tested;

b Tested only in Apis cerana;

c Tested only in Apis florea;

d Van den Heever et al. (46) found no effect;

† *Bee mortality varied between treatments and compound concentrations; +β-phellandrene, eucalyptol, and α-terpineol*.

## Small Molecules

Studying the biological activity of small molecules presents a promising strategy for discovering a new anti-*Nosema* therapy. A novel *N. ceranae* cell culture procedure can be adapted to a 96-well microplate format, thus making high and medium throughput drug screening assays on *N. ceranae* feasible and efficient (44). Two nitroimidazole compounds (metronidazole and tinidazole) that greatly reduce *N. ceranae* viability *in vitro* with low cytotoxicity have been identified using this method (44). While the method described could be useful in screening a high number of molecules, the likelihood of applying these two compounds to apiarian medicine is low, as nitroimidazole compounds are unapproved by many countries for use in treating food animals (MRL; Commission Regulation, EU, 2010, no. 37/2010). A more current study tested both *in vitro* and *in vivo* activity of porphyrins against *N. ceranae*. Porphyrins are aromatic heterocyclic compounds conserved in nature that are involved in many biological processes, including oxygen transport and photosynthesis (45). Treating spores and infected bees with select non-metallated porphyrins [PP(Asp)_2_ and TMePyP] in sugar syrup significantly reduced microsporidian viability *in vitro*, decreased infection levels of inoculated honey bees by up to 5 fold, and increased bee survival [PP(Asp)_2_ only; (45)]. The investigators postulate that porphyrins may act on the cell wall or membrane, as deformities in spore exosporium layers were observed following spore pretreatments.

It is noteworthy that inhibition of the enzyme methionine aminopeptidase type 2 (MetAP2) is fumagillin's proposed mechanism of action against *Nosema* (37, 42, 46). MetAP2 specifically catalyzes the cleavage of initiator methionine on the N-terminal of newly-synthesized proteins, serving an important function in post translational modification (69). Although most animals express two functional MetAP isoforms (MetAP1 and MetAP2), microsporidia express only MetAP2 (69). Therefore, use of MetAP2 antagonists to target *Nosema* could be a viable strategy for controlling nosemosis in honey bees. Van den Heever et al. (46) recently screened several analogs of fumagillin (and other commercially-available compounds) in cage experiments and observed a significant decrease in *N. ceranae loads*. Although the authors demonstrate efficacy, none of the compounds tested were as effective as fumagillin at eliminating *N. ceranae* spores. Given the tight regulation on use of antibiotics in food animals, precautions should be taken in the development and approval of novel MetAP2 inhibitors for apiary medicine.

Repurposing currently-used honey bee medications may be another favorable strategy for controlling *N. ceranae*. Oxalic and formic acid, which are used as miticides by beekeepers to suppress varroa mites (devastating honey bee ectoparasites), have inactivated *N. ceranae* in both laboratory and field trials (47, 48). In an indoor fumigation experiment, Underwood and Currie (47) noted that formic acid fumigation lowered *Nosema* spore loads in colonies over the course of 1 year. Indoor fumigation treatments, while potentially efficacious, are probably not cost-effective nor practical on a commercial beekeeping level. A more practical method would be to implement natural fumes or vapors created by solubilized or liquid compounds, similar to topical varroa mite treatments [formic acid, oxalic acid, etc.; (36)]. Nanetti et al. (48) applied this concept, finding oral oxalic acid (0.25 M in sugar syrup) treatments in caged bees and topical treatments in field trials to significantly decrease the rate of infection and increase colony survival compared to controls. These findings are notable since varroa mites are usually controlled by topical, non-oral treatments (36). Use of a topical, fume-generating treatments may have advantages over oral medications since delivery is not subject to variable feeding behavior (storing, hording, poor winter feeding, etc.). Other phenolic compounds commonly used to combat varroa mite infestations, particularly resveratrol and thymol (35, 36), are effective at inhibiting *N. ceranae* in oral preparations (46, 49, 50). Larger colony survival surveys and toxicity studies are still required for these treatments. Together, these studies suggest that certain organic acids, phenolics and other compounds impede *N. ceranae* viability and may have duel treatment applications given the continuing need for miticides in apiarian medicine.

Most experimental *Nosema* treatments target spores in the honey bee digestive tract, leaving viable spores in hive structures, nectar combs, and feces free to infect or re-infect naïve or treated animals. Consequentially, future studies could more thoroughly investigate dosage and synergy between treatment types that target spores in various life stages. Pairing fume-generating topical treatments (e.g., oxalic acid-soaked pads) with oral medications, for example, may kill both reproducing and free spores in the hive environment, while also controlling varroa mites.

## RNA Interference

Investigating RNAi may be of use in the discovery of novel targets and treatments for honey bee *N. ceranae* infections. RNAi is a post transcriptional gene silencing mechanism that is driven by double-stranded RNA (dsRNA) binding to homologous transcript sequences of a target gene (70). Moreover, RNAi is a natural anti-infective mechanism of the honey bee immune response (71). RNAi is currently being explored for therapeutic activity in human medicine and pesticide activity in agriculture (72–74). Inhibition of varroa mites and several RNA viruses infectious to honey bees has also been accomplished by RNAi (75–82).

Previous work has applied RNAi of *Nosema* nucleotide transporter genes to control nosemosis in honey bees (51). The genome of *N. ceranae* has been previously sequenced with high specificity of ATP/ADP transporter isoforms (51). ATP/ADP transporter proteins are important for maintaining microsporidian physiological processes (83). This study demonstrated that target transcript levels and host spore loads decrease when honey bees ingest daily doses of synthetic dsRNA (in sugar syrup) specific to *N. ceranae* ATP/ADP transporters (51). In addition, worker bee responsiveness to sucrose (quantified by measuring the proboscis extension reflex), which increases during *N. ceranae* infections (84), decreases at low sucrose concentrations following RNAi treatment. A more recent study used RNAi to lower expression of polar tube protein 3 (ptp3), a protein essential for sporoplasm injection and microsporidian cellular invasion (32, 33, 52). When *ptp3* is knocked down via ingestion of dsRNA, host spore loads decrease, several antimicrobial peptides (Abaecin, apidaecin, hymenoptaecin, defensin-1) normally downregulated by *N. ceranae* infections (85, 86) are upregulated, and survival is significantly prolonged (52). The *N. ceranae Dice*r gene has also been identified as a possible RNAi target and may have implications toward minimizing infectivity when knocked down (87, 88).

In addition to targeting *N. ceranae* genes specifically, RNAi has been employed to reduce expression of negative regulators of the honey bee immune response (53). It has been shown that *N. ceranae* infections downregulate several immune genes and upregulate the naked cuticle gene (*nkd*), an antagonist of the WNT pathway and an important regulator of immune function (53, 89–91). Following ingestion of dsRNA targeting *nkd*, lower infection levels and increased immune expression and survival are observed in bees (53).

RNAi-mediated knockdown of genes important for *N. ceranae* viability or honey bee immunoregulation may have the potential to control *Nosema* disease. Nevertheless, several obstacles should be considered when evaluating the feasibility of RNAi-based bee medications. Oral delivery of dsRNA to honey bees may lower RNAi efficiency and stability, as digestive enzymes and gut pH can rapidly metabolize and alter the drug sequence before delivery to target mRNA (92). A synthetic coating (e.g., nanoparticle/liposome) may provide protection but could also increase production costs (92). Off target and non-specific effects of RNAi are another major concern in agriculture that will likely slow the approval of RNAi-based treatments for apiarian medicine. Although many applications of RNAi have been thoroughly researched, no RNAi-based drugs or pesticides have been approved for agricultural use. The only EPA-approved application of RNAi in agriculture is a strain of corn genetically-modified to express rootworm-targeting dsRNA (93). RNAi has certainly demonstrated value in identifying potential *N. ceranae* drug targets, but more research is needed in order to show that RNAi as a therapy in the beekeeping industry is safe, cost-effective and practical.

## Extracts and Natural Supplements

The effects of organic extracts and natural supplements on *N. ceranae* infections have been extensively explored. Such treatments are attractive to agriculturalists and environmentalists since toxicity is less of a concern compared to other chemical treatments. Indeed, various organic and aqueous natural product extracts have been shown to increase bee survival and lower spore loads following oral treatment (54, 56–61). It should be noted that ethanolic propolis extracts evaluated by Yemor et al. (60) and Suwannapong et al. (61) were tested in different bee species (*Apis florea* and *Apis ceranae*) which are globally less important pollinators than honey bees. Natural compounds, particularly flavonoids, have been detected in several plant extracts displaying anti-microsporidian activity in honey bees, although flavonoids have not been confirmed to be the source of this activity (59). Bravo et al. (58) reported *in vivo* anti-*Nosema* activity similar to fumagillin in essential oil (EO) hydrodistillation extracts from *Cryptocarya alba* leaves. Select monoterpenes (β-phellandrene, eucalyptol and α-terpineol) detected in the extract also inhibited *N. ceranae* (58). Interestingly, the chemical make-up of crude extracts is usually complex and often unknown; for example, plant and propolis extract purity and molecular composition vary greatly between extract batches and sources [e.g., geographical region; (94)]. However, these variations may have a significant effect on product potency. Production of extracts and supplement-based bee treatments must be highly standardized in order to provide confidence of efficacy in the field.

Commercial supplements have been studied for activity against nosemosis. Colonies supplemented with gastric pentadecapeptide BPC 157, a well-studied antiulcer peptide, demonstrate increased worker bee colony population, lower spore counts, and limited lesions to the midgut of infected bees (55). The experiment was not of sufficient length to show an effect on colony survival (55). Additionally, a dietary amino acid and vitamin complex called BEEWELL AminoPlus decreases spore loads and protects honey bees from immune suppression by upregulating expression of antimicrobial peptides (62). Preliminary data suggest that a commercial phytopharmacological supplement, Nozevit®, may improve bee health by decreasing colony spore loads (63). Further investigation and a larger sample size is needed in order to confirm these results, as van den Heever et al. (46) reported no effect of Nozevit® in cage trials. A 2-year survey of the seaweed-based supplement HiveAlive™ reported a decrease in colony spore loads and an increased hive population relative to controls following administration of two biannual treatments (64). Surprisingly, survival was not commented on in this study, notwithstanding the authors account for colony mortality in their analyses of colony strength (64).

Although certain natural extracts and commercial supplements have shown efficacy against *N. ceranae*, there are other natural product supplements advertised as anti-infective that do not have any beneficial effects on honey bees infected with *N. ceranae*. Nosestat® and Vitafeed Gold® were evaluated in a field trial and found to have no impact on colony productivity and *Nosema* spore levels (95). ApiHerb® and Nonosz® are also sold to improve bee health and perhaps treat nosemosis, but additional research and more scientific evidence is needed in order to support claims of efficacy (95). Evidently, beekeepers should be cautious about which supplements and extracts they select for treating *N. ceranae* infections.

## Microbial Supplements

Administering microbial supplements may have positive impacts on honey bee health and impair *N. ceranae* viability. Baffoni et al. (67) suggest that supplementing the honey bee diet with strains of *bifidobacteria* and *lactobacilli*, which secrete antibiotic metabolites, lower *N. ceranae* spore levels. This work adds to previous studies indicating that organic acids and other metabolites (e.g., surfactin) produced by bacteria reduce bee mortality and *N. ceranae* loads when fed to honey bees (65, 66). Other bacterial strains and probiotics (*Parasaccharibacter apium, Bacillus* sp., Bactocell®, and Levucell SB) have been shown to improve survival of infected bees but not decrease spore loads (68). A successful anti-*Nosema* treatment should improve bee health and lower infection levels. Select probiotics, prebiotics, and pollen substitutes may actually exacerbate infections and increase bee mortality (38, 96–98). Alternative methods previously described are likely more promising and applicable to beekeeping than microbial supplements.

## Conclusion

Recent laboratory and field studies report encouraging results suggesting that single compounds, RNAi, natural extracts and supplements may impair *N. ceranae* and improve colony health. It is important that researchers continue to test novel agents for anti-microsporidian activity against nosemosis.

## Author Contributions

AB conceived, designed, and wrote the manuscript.

### Conflict of Interest Statement

The author declares that the research was conducted in the absence of any commercial or financial relationships that could be construed as a potential conflict of interest.
